# From Albania to Zimbabwe: Surveying 10 Years of Summer Field Experiences at the Rollins School of Public Health

**DOI:** 10.9745/GHSP-D-16-00262

**Published:** 2017-09-27

**Authors:** Evelyn L Howatt Donahoe, Roger W Rochat, Deborah McFarland, Carlos del Rio

**Affiliations:** a Hubert Department of Global Health, Rollins School of Public Health, Emory University, Atlanta, GA, USA.

## Abstract

The objective of this article is to describe summer field experiences at the Rollins School of Public Health. An online survey was conducted among Master of Public Health students returning from summer field experiences. We used printed reports from 2004–2012 and original survey data from 2010–2013 to perform a trend analysis using correlation analysis and linear regression. We found that our students have worked for more than 300 organizations in 84 countries. The average cost of a summer field experience fluctuated around US$3,500, with students receiving an average of US$2,180 in funding. About 50% of students conducted human subjects research. This survey was used to improve student practical experiences through information sessions for students and feedback to key constituents, including the Emory Institutional Review Board and the Emory Travel Clinic.

## Abstract (precis)

Since 1985, students from the Rollins School of Public Health have worked for more than 300 organizations in 84 countries. The students indicated key benefits of applying public health course work in real-world settings and gaining skills, including cultural competency, leadership, teamwork, communication, and program implementation. They also experienced challenges related to health, safety, and support.

## INTRODUCTION

All Master of Public Health (MPH) students attending an accredited school are required to complete a practicum of at least 200 hours before graduating. Increasing numbers of these students have sought opportunities abroad to fulfill this requirement.^1^ International field experiences are often expensive and can require additional effort on the part of students to find and gain access to these opportunities. This has been the case for many MPH students, both those in global and non-global programs, at the Rollins School of Public Health (RSPH) of Emory University. Although RSPH has never had a formal program that links students to international practicum opportunities, as early as 1985, students used the summer between the first and second years of the MPH program to engage in applied public health work across the globe.

**FIGURE 1:**
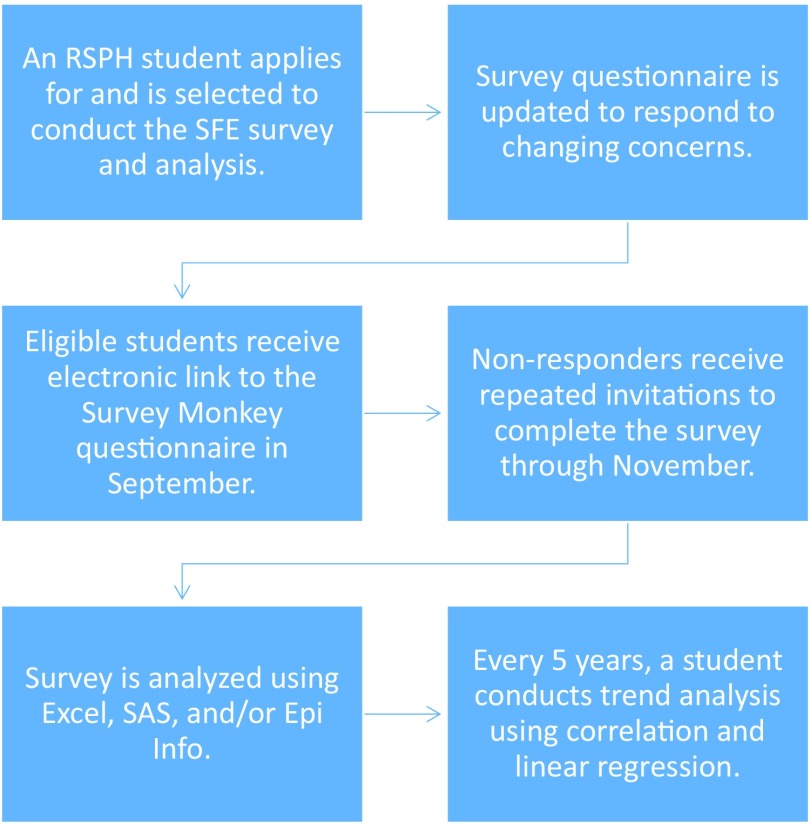
Annual RSPH Summer Field Experience Survey Process Abbreviations: RSPH, Rollins School of Public Health; SFE, summer field experience.

By 2004, anecdotal feedback from students indicated that they did not feel prepared for the public health work they were engaging in over the summer.^2^ In response, the Hubert Department of Global Health created an annual survey to evaluate their summer field experiences (SFE), which became known as the SFE survey (Figure 1). Initially, the survey asked questions related to perceptions of student preparedness and how to improve the quality of these global public health work experiences. It later expanded to ask additional questions related to Institutional Review Board (IRB) processing times, health and safety issues, and finances. The survey results continue to be used to describe how students find international summer opportunities. We use it to align our educational program with the needs of students by enhancing the curriculum; advising students; deciding how to allocate funds; improving IRB training, consultation, and turnaround time; and improving Emory Travel Clinic policy and training, among others.^2^ The school currently asks all public health students planning international practicums, regardless of funding, to attend 5 hours of pre-departure safety and security training, 1 hour per day during a dedicated week in the spring semester. The agenda includes preparation, travel policies, physical and emotional health, personal safety, and sexual harassment/assault.

## METHODS

Since 2004, the Hubert Department of Global Health at RSPH has been conducting an annual SFE survey of students. Specifically, at the beginning of the second semester each year, the Director of Graduate Studies in the Hubert Department of Global Health and a selected student use Survey Monkey to design and conduct the annual survey of second-year students to evaluate their activity during the summer between the second and third semesters. Respondents include all public health students in global health academic programs; all public health students receiving Global Field Experience funds, regardless of academic department; and all students funded by the Emory Global Health Institute for international, multidisciplinary public health team projects (often used by graduate students in public health for their obligatory public health practicum).

The content of the questionnaire varies from year to year, but we consistently seek to evaluate what makes a satisfactory, successful, healthy summer field experience; how students obtain these field experiences; and the expenses and sources of funding support. We seek to make the final evaluation reports each year readily accessible to all students.

In 2004, the Emory IRB reviewed the survey and determined that it is program evaluation, not human subjects research. The survey is confidential, but not anonymous.

For the purpose of this article, we reviewed the printed survey evaluation reports from 2004 to 2012, along with the raw survey data from 2010 to 2013. We describe trends and patterns based on correlation analysis and linear regression.

## RESULTS AND DISCUSSION

**FIGURE 2:**
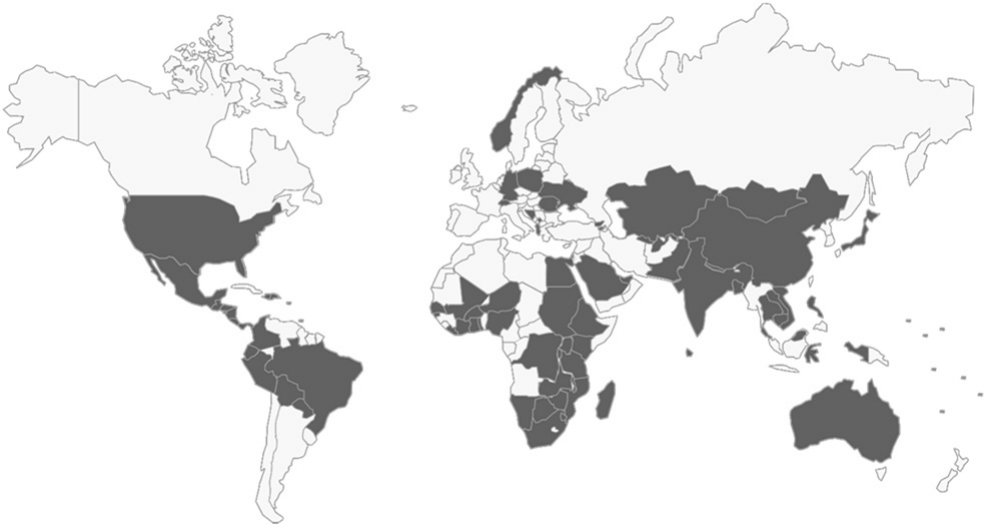
Locations of RSPH Summer Field Experiences, 1985–2014 Note: Partial data were used for years prior to 2009. Data were not available for 2010.

Between 2004 and 2013, a total of 1,048 students completed the survey, with an overall response rate of 89%.^2^^–^^11^ The number of students responding increased from 68 in 2004 to 147 in 2013. The students have worked in very diverse locations on a range of health-related issues with a number of host organizations (Figure 2). An estimated 84 countries and 370 different organizations have hosted RSPH students since 1985.

An estimated 84 countries and 370 different organizations have hosted RSPH students since 1985.

### Practicum Opportunities and Selection

Of key importance to this evaluation is recognizing the ways in which students identified and accessed SFE opportunities. Most students identified a viable practicum opportunity during the first or second semester through faculty advisors, networking with second-year students, or during an annual practicum fair in late October. First-year students were able to learn about SFEs during their first semester from the presentations of second-year students. In 2013, half of the students who went abroad said they identified their summer practicum through their own personal and work contacts or other students. The timing of applications depended on the size and scope of the project. For students choosing to develop multidisciplinary projects that involved students from at least 3 Emory schools, proposals were due by the end of January. Students applying for Global Field Experience (GFE) funding had to submit their individual proposals by the end of February.

While this autonomy likely contributed to the great diversity in experiences, it may also have drawbacks. Each year, a few students have their experiences cut short because they discover upon arrival that the host organization is not prepared or the student has not communicated sufficiently with the organization. In many cases, the student is able to find a second opportunity; however, this is not always the case. For those using the SFE as a public health practicum, the field supervisor must approve the project in advance and assess progress midway and at the end of the project. This process helps reduce the number of student–host miscommunication issues.

### Funding of Practicums

No matter how students identify their practicum opportunity, several mechanisms to assist with funding for SFEs outside the United States are available through the university. Based on survey responses, each year about 60% of students who had SFEs outside the United States received financial support from the GFE, which provides small grants from Emory-endowed funds. GFE grants require a proposal submission, and the amount of funds allocated depends on proposal quality and time of submission. Students who receive GFE funds are required to present their work at the end of the summer.

The second most common source of funding is the Emory Global Health Institute (EGHI), which sponsors about 25% of students with SFEs outside the United States. EGHI-sponsored students are required to work in multidisciplinary teams that include people outside of the school of public health. In addition, the Global Elimination of Maternal Mortality from Abortion (GEMMA) fund supports about 10 students each year in practicums related to reproductive health and abortion.

Despite these funding options, most students pay for at least part of their experience out of pocket: the average cost of an SFE outside the United States is about US$3,500, while the average amount of funding received per student is about US$2,180.^13^^–^^17^ About a fifth of students have fully funded summer experiences, and most pay less than US$2,000 out of their own pockets. The combination of the financial assistance available and the autonomy with which students identify and set up their summer experiences are likely to contribute to the great diversity in experiences: not only have students worked on every continent, except Antarctica, and worked with 370 organizations, but most were the only students to ever work with their organization. In fact, this was the case for 75% of the students.

75% of the students were the only students to ever work with their summer-experience organization.

This diversity is both a strength and a weakness. Students are able to pursue practicum opportunities that are most in line with their interests and career goals. Subject areas have included nutrition, infectious disease, refugee health, homelessness, faith and health, communications, technology in public health, addiction, geriatric care, one health, health systems, and water, sanitation, and hygiene (Box). However, since most organizations have only hosted an RSPH student once, few hosts have provided continuity in sequential years. Notably, a few organizations consistently provide practicums: each year about 7% of students worked with the U.S. Centers for Disease Control and Prevention (CDC), 3% with CARE, 2% with the Rwanda-Zambia HIV Research Group, and 2% with the Center for Global Safe Water.

BOX. **Four Case Studies of Rollins School of Public Health International Practicums**

**Kyu Han Lee: Antimicrobial Resistance in Abu Dhabi, United Arab Emirates**

**Figure:**
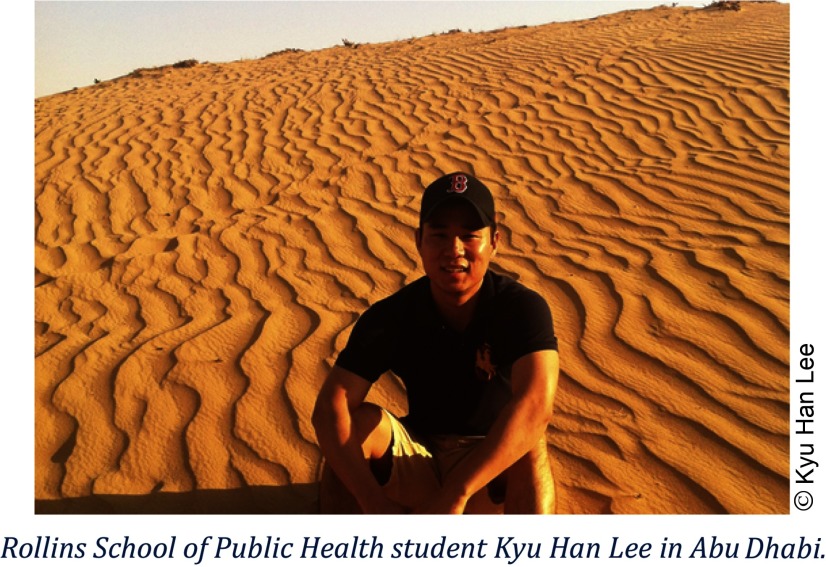
Through retrospective data analysis, Kyu Han Lee identified several potential multidrug resistant outbreaks previously undetected by infection control personnel. Organization: Health Authority, Abu Dhabi.

**Anna Fulton: An Oral Cholera Vaccine Intervention in Mae La Refugee Camp, Thailand**

**Figure:**
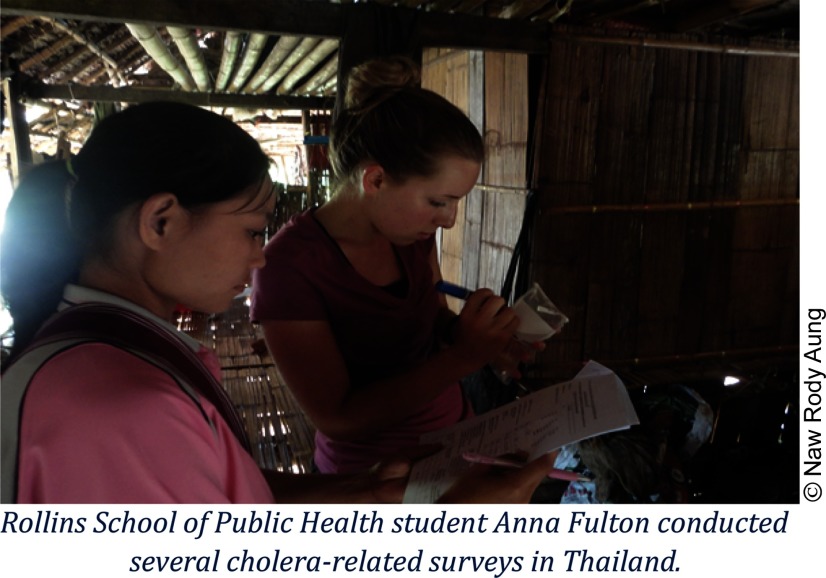
Anna Fulton conducted a Knowledge, Attitudes, and Practice survey after a cholera vaccine intervention; conducted microbial testing on household drinking water samples; and conducted a census verification in a camp of 46,000 refugees leading to more accurate follow up of vaccinated individuals. Organization: U.S. Centers for Disease Control and Prevention (CDC).

**Grayson Privette: Emergency Preparedness and Response: Mapping Guatemala's Health Infrastructure**

**Figure:**
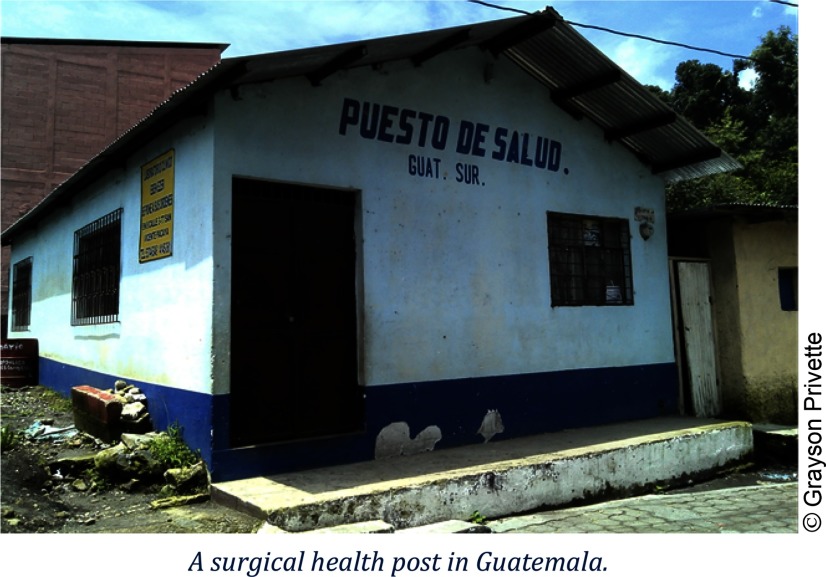
Grayson Privette created a surge capacity tool, which was used to assess the characteristics, capabilities, and spatial distribution of health care infrastructure for emergency preparedness and response. Organization: CDC.

**Lisa Sthreshley: Cultural, Social, and Economic Factors That Influence Household Adoption of Fuel Efficient Stoves in Kinshasa, Democratic Republic of the Congo**

**Figure:**
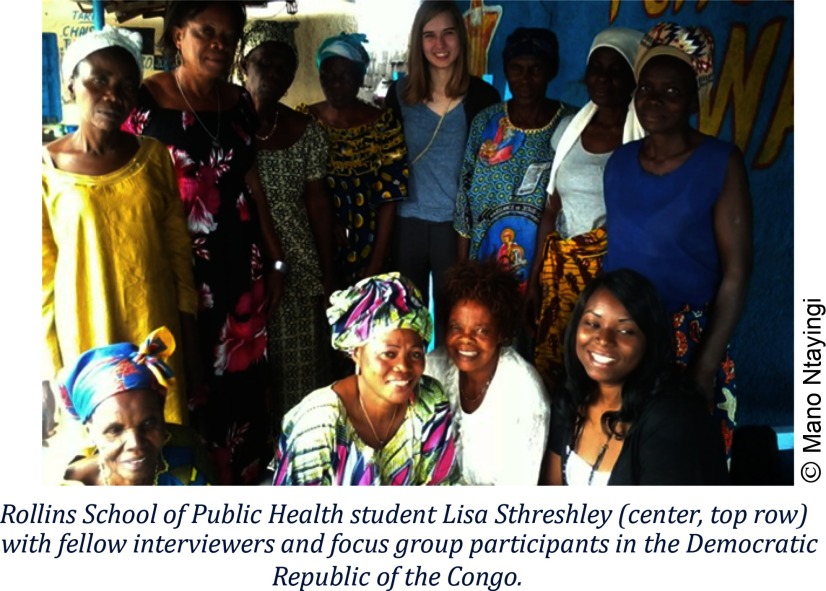
Lisa Sthreshley conducted an evaluation of Top-Lit Up Draft stoves designed to reduce indoor air pollution in the Democratic Republic of the Congo. Results of the analysis led to improvements in the design of the stove. Organization: IMA World Health.

### Benefits and Challenges of the Practicum Experience

For students, perhaps the greatest benefit of the SFE is the opportunity to apply their public health course work in a real-world setting. Survey respondents frequently cited the opportunity to practice both qualitative and quantitative methods as beneficial for future careers:

*Finally putting my qualitative research skills to work … I gained many skills from tool development to conducting actual qualitative research and fieldwork.* –RSPH student completing an SFE in Paraguay, 2013

In addition, students commonly reported that their field experience increased their cultural competency, leadership, coordination, language, teamwork, communication, interpersonal, and program implementation skills, which are often more difficult to teach in a classroom setting.

*I gained many cross-cultural skills including communication, increased flexibility … the professional and personal development from the experience is invaluable.* –RSPH student completing an SFE in Armenia, 2013

Students have also shared their struggles, including difficulties related to health, safety, and support. By 2006, enough students had written that they felt unsupported by RSPH during their practicum that the Likert-scale question “How supported did you feel?” in relation to school support was added to the survey. In response, 60% of students said they felt academically supported, while only 45% said they felt psychologically supported during their SFE. The issues of support, health, and safety continue to be important to both the students and the school.

Between 2006 and 2014, 10 students reported being sexually or physically assaulted during their international practicum. Subsequent data collection showed that in 2015 more students reported sexual assault than ever before (although that data are not represented in this paper). Lack of disclosure or reporting by students, faculty, and others to protect the privacy of students may lead to an undercount of these events.

About half of the students reported experiencing at least one 1 health problem during the summer, with several students each year reporting serious events requiring hospitalization or evacuation from the host country. In these cases, Emory's student insurance has been able to assist with organizing and paying for the evacuation services.

Survey questions that ask about assault, harassment, and illnesses, such as diarrhea, respiratory infections, and mental health problems, have helped the Emory Travel Clinic and Emory University design a health and safety training for students before their departure. The school currently asks all public health students planning international practicums, regardless of funding, to attend 5 hours of pre-departure safety and security training, 1 hour per day during a dedicated week in the spring semester. The agenda includes sessions on preparation, travel policies, physical and emotional health, personal safety, and sexual harassment/assault. Student comments on the 2013 survey prompted the Travel Clinic to provide more private spaces for travel consultations.

### Institutional Review Board Processes

All students are required to have Collaborative Institutional Training Initiative (CITI) certification and to obtain Emory IRB review for human subjects research. Each year between 32% and 71% of SFE students engage in human subjects research. However, how the IRB processed and classified applications changed over the duration of the study period. For example, during 2005–2007, the IRB expedited most SFE proposals. In contrast, after 2007, the IRB classified most proposals as exempt. In 2013, of 56 proposal submissions, 28 were exempt, 13 expedited, and 6 underwent full review.^18^^–^^22^

Students are often required to submit their proposals to multiple IRB or ethics review committees, depending on their research topic, location, or host organization. Proposals may need to be submitted to not only Emory but also the host organization and/or host government IRB-type mechanisms. For example, in 2013, 62% of students working outside the United States received approval from an external IRB, in addition to the Emory IRB. Moreover, 58% of students provided training on ethics, privacy, confidentiality, or informed consent to project staff during their SFE.

**FIGURE 3:**
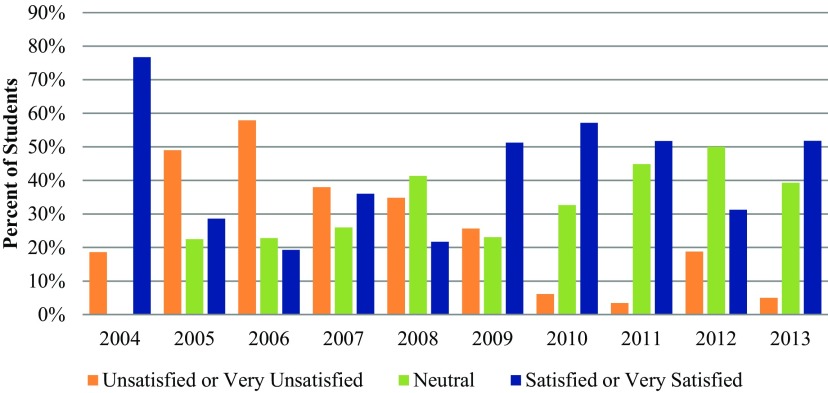
Proportion of Students Who Are Satisfied With the Institutional Review Board Review Process, 2004–2013 Note: Data from 2010 to 2013 reflect only SFEs based outside of the United States. A mistake in the survey questionnaire for 2013 did not allow students to select **“**very unsatisfied**”** as an option. Data include students who submitted to the IRB themselves and students whose IRB submission was completed for them by their host organization or a faculty member. In 2004, students were not able to select a **“**neutral**”** option. Source of data: References 8-11, 18-22.

In 2006, the student surveys documented long delays in IRB staff responses, such as an average of 32 days from protocol submission to receiving a response, problems communicating with IRB staff, issues related to international research, and problems with protocol review. That survey led to the IRB process providing faculty and students with clearer instructions about protocol submissions, how to obtain appropriate informed consent in different settings, how to present appropriate research methods and appropriate data security, and what confidentiality steps required for working in international settings. In addition, IRB staff clarified informed consent guidelines for international populations. Concurrently, our department began requiring that all new students complete the CITI certification before enrolling. These changes led to improved student protocol submissions and IRB responsiveness (Figure 3).

### Prior Coursework and Experience

Finally, questions related to which coursework most prepared students for their fieldwork have assisted students, mentors, and academic advisors to determine the best courses to take prior to their practicum. About a third of students said their coursework prepared them “a lot” for their practicum, while a fifth said it prepared them only “a little” or not at all. Students were most likely to list methods courses—such as survey design and qualitative methods—as the most useful and were also likely to list these among skills they wished they had before their practicum. Students also reported that prior international and research experiences helped them as much as the academic courses.

### Limitations

The survey is limited to student observations. At present, we lack the resources to conduct a systematic evaluation of the value of student projects to the host organizations. By challenging students to have expertise and resources for useful and doable projects, we try to ensure the SFE is beneficial to the host community. Host organizations occasionally offer jobs to students, co-author publications, or use the projects to further their organizations' missions. In 2013, 83% of students reported they debriefed their host organization.

## CONCLUSION

Using SFE survey data to monitor, evaluate, and report on student activities has been an invaluable experience for the Department of Global Health at RSPH. Because of the amount of autonomy students have to set up their practicums, without this survey, we would have a much more limited picture of the work students do over the summer, the challenges they encounter, and the preparation they require. We have learned the importance of the funding provided to students, and how it opens up opportunities for many of them to work for small organizations in developing nations. We have also gained insight into the physical and emotional challenges that students face during international practicums. Having adequate insurance in order to assist students requiring evacuation for health or security reasons has proven essential.

Because of the amount of autonomy students have to set up their practicums, without this survey, we would have a much more limited picture of the work students do over the summer, the challenges they encounter, and the preparation they require.

The early surveys were intended to provide guidance for students, faculty, and administrators of the school's global programs. Serendipitously, in 2006, we found that students' documentation of challenges with the IRB process was also useful for the university.^4^ In 2013, an MPH student conducted his master's thesis on how to improve the use of SFE information. After focus groups and individual interviews, he recommended the provision of 9 specific short reports with relevant information for the following designated recipients: (1) The Director of Graduate Studies, Global Health Department; (2) all RSPH students; (3) Associate Directors for Academic Programs (ADAPs); (4) Career Services; (5) faculty and staff; (6) Emory IRB; (7) Emory Travel Clinic; (8) Health and Safety for GFE Committee; and (9) Highlights for Global ADAPs.^10^

Although we do not provide students with practicum opportunities, we have been able to help them identify networks to find them, select appropriate coursework, provide some funding, and give health and safety information before their departure (Worrell MC, SFE Organizations Google sheet, 2013; Worrell MC, Peters K, Countries SFE Surveys Google sheet, 2013).^3^^–^^22^ While we have not yet found solutions to all the problems raised, the survey has allowed us to be more responsive to changing concerns and needs among the student body and to document the growth of interest in global field experiences. We hope that the experience and results shared here can assist other schools of public health and educators as they seek to develop and improve practicum programs as well.

## Notes

**Peer Reviewed**
